# New Applications of Synthetic Biology Tools for Cyanobacterial Metabolic Engineering

**DOI:** 10.3389/fbioe.2019.00033

**Published:** 2019-02-27

**Authors:** María Santos-Merino, Amit K. Singh, Daniel C. Ducat

**Affiliations:** ^1^MSU-DOE Plant Research Laboratory, Michigan State University, East Lansing, MI, United States; ^2^Department of Biochemistry and Molecular Biology, Michigan State University, East Lansing, MI, United States

**Keywords:** cyanobacteria, metabolic engineering, synthetic biology, genome scale models, photosynthesis

## Abstract

Cyanobacteria are promising microorganisms for sustainable biotechnologies, yet unlocking their potential requires radical re-engineering and application of cutting-edge synthetic biology techniques. In recent years, the available devices and strategies for modifying cyanobacteria have been increasing, including advances in the design of genetic promoters, ribosome binding sites, riboswitches, reporter proteins, modular vector systems, and markerless selection systems. Because of these new toolkits, cyanobacteria have been successfully engineered to express heterologous pathways for the production of a wide variety of valuable compounds. Cyanobacterial strains with the potential to be used in real-world applications will require the refinement of genetic circuits used to express the heterologous pathways and development of accurate models that predict how these pathways can be best integrated into the larger cellular metabolic network. Herein, we review advances that have been made to translate synthetic biology tools into cyanobacterial model organisms and summarize experimental and *in silico* strategies that have been employed to increase their bioproduction potential. Despite the advances in synthetic biology and metabolic engineering during the last years, it is clear that still further improvements are required if cyanobacteria are to be competitive with heterotrophic microorganisms for the bioproduction of added-value compounds.

## Introduction

Over 1 billion years ago, cyanobacteria began to drive the rise of oxygen in Earth's atmosphere, setting the stage for the evolution of new forms of complex, multicellular organisms (Shih, 2015). More recently, these photosynthetic prokaryotes have come under increasing scrutiny for their potential to sustain the lifestyle of complex eukaryotic lifeforms, this time as potential bioproduction hosts. Cyanobacteria can efficiently harvest CO_2_ as a carbon source, powering their metabolic processes by absorption of sunlight, the most abundant form of renewable energy. As fast-growing, and relatively simple bacteria, cyanobacteria hold the promise of being an ideal chassis for ambitious metabolic engineering projects. In recent years, cyanobacterial potential is being unlocked through the development of an increasing number of molecular tools (Berla et al., 2013; Carroll et al., 2018; Sun et al., 2018). These advances have been coupled with increasing capacities to manipulate endogenous genetic sequences and transfer exogenous DNA into multiple cyanobacterial strains (Berla et al., 2013; Cassier-Chauvat et al., 2016). Moreover, due to advances in sequencing technology, the genomes of >270 cyanobacterial species have been sequenced (Fujisawa et al., 2017), greatly facilitating the application of systems-level techniques, such as transcriptomics and proteomics. These technological improvements in the manipulation of cyanobacteria are set against the backdrop of increasingly refined tools that have been developed in the fields of metabolic engineering and synthetic biology, potentially setting the stage for cyanobacteria to significantly contribute as crop species in the Twenty-first century.

Metabolic engineering can be defined as the practice of optimizing genetic and regulatory processes in cells to increase production of certain metabolic substances (Kumar and Prasad, 2011). This methodology has been used in cyanobacteria as well as other bacteria, to expand the list of products that they are able to make and increase production efficiency (Angermayr et al., 2015; Lai and Lan, 2015; Carroll et al., 2018). Traditional approaches to improve production of a specific compound were based on random mutagenesis and selection or the targeted introduction of individual genes and mutations, which requires considerable time to design and implement (Kumar and Prasad, 2011). Recently, systems metabolic engineering has emerged as new methodology for solving these issues (Nogales et al., 2013). It is based on the use of mathematic models to simulate and predict behaviors that emerge in complex systems, and has been used extensively for the improvement of microbial production (Lee et al., 2011).

In parallel, synthetic biology principles promote a bottom up approach to design biological systems, recombining defined parts or modules to restructure existing systems or build new pathways *de novo* (Sengupta et al., 2018). It is based on the construction of intricate biological systems using standardized, well-characterized, and interchangeable biological parts, or “modules” (Cheng and Lu, 2012). Collectively, these modules form “toolboxes” of components that can be used to modify an organism, including a catalog of characterized biological parts [e.g., promoters, ribosome binding sites (RBSs), riboswitches, terminator libraries], standardized methods for the assembly and manipulation of genetic components, and predictive models designed to facilitate pathway optimization (Gordon et al., 2016). Ideally, truly modular biological parts would be characterized in a manner that would allow researchers to accurately predict how they will function in the context of an interconnected system (Pasotti et al., 2012). In practice, it remains difficult to characterize parts in a manner that is completely transferrable to other organisms (Sengupta et al., 2018), because they are typically characterized by a specific research group in particular environment (Decoene et al., 2018). Another challenge to the transfer of parts is that biological components are usually non-orthogonal and may interact with genes, proteins, and metabolites of the chassis organism (Fu, 2013), or have their function influenced by host pathway components (Wang et al., 2013). Still, largely by using specific parts and strategies developed in model heterotrophic organisms, useful synthetic biology components have been recently adapted and debugged for use in cyanobacteria.

This review focuses on recent engineering tools and strategies developed for metabolic engineering of cyanobacteria as hosts for generating added-value compounds while using solar energy and CO_2_ as inputs. We discuss the current challenges and opportunities for the application of synthetic biology principles in cyanobacteria. Finally, we highlight the potential for genome-scale models as tools to assist cyanobacterial engineering.

## Cyanobacteria as Host for Biomolecule Production

Cyanobacteria stand out as one of the most promising candidates as hosts for bioproduction (Knoot et al., 2018). Because cyanobacteria utilize solar energy to fix carbon dioxide, a greenhouse gas, and can convert these reduced carbon products into valuable metabolites (Lau et al., 2015), they are especially attractive in an era where sustainable biotechnological processes are of increasing importance (Ruffing, 2011). Additionally, cyanobacteria possess a number of advantageous features relative to other photosynthetic organisms. In comparison to eukaryotic algae and plants, cyanobacteria are more genetically tractable (Parmar et al., 2011; Lau et al., 2015), grow more rapidly, and can achieve higher efficiencies of solar energy capture and conversion (Dismukes et al., 2008; National Academies of Sciences, 2018). Furthermore, cyanobacteria can be cultivated without the need for arable landmass or potable water supplies (Nozzi et al., 2013) and can potentially even degrade aquatic pollutants, such as aromatic hydrocarbons (Ellis, 1977; Cerniglia et al., 1979, 1980; Narro et al., 1992) and xenobiotics (Megharaj et al., 1987; Kuritz and Wolk, 1995) to remediate contaminated water supplies. Yet, relative to other photosynthetic organisms, especially plants, cyanobacteria are currently not used in many scaled agricultural or biotechnological applications. The underutilization of cyanobacteria stems partially from their relative novelty as crop species. Whereas, technologies for cultivating, harvesting, and breeding plants have been under extensive development for many millennia, comparable research to improve the prospects for cyanobacterial cultivation has largely been pursued only since the 1970s (Sheehan et al., 1998).

Cyanobacteria make a number of compounds that are comparable to food, fiber, and fuel products routinely acquired from plants, although cyanobacterial strains have not been as extensively modified to improve their compatibility for scaled cultivation. Like many genera of eubacteria, cyanobacteria can synthesize polyhydroxyalkanoates, a thermoplastic class of biodegradable polyesters that includes polyhydroxybutyrate (Quintana et al., 2011). Many cyanobacterial strains also produce a wide spectrum of secondary metabolites with high-value commercial properties, such as pigments, vitamins, amino acids, macrolides, fatty acids, lipopeptides, and amides (Lau et al., 2015). In total, cyanobacteria are estimated to have the capacity to produce around 1,100 secondary metabolites (specific cyanobacterial bioproducts are beyond the scope of this article, but are reviewed comprehensively in Dittmann et al., 2015; Salvador-Reyes and Luesch, 2015; Xiong et al., 2015, 2017). Beyond natural metabolites, engineering efforts have been used to redirect the metabolism of model cyanobacteria toward biosynthesis of heterologous bioproducts including alcohols, fatty acids, hydrocarbons, fatty alcohols, olefins, organic acids, sugars, and polyols (engineered cyanobacterial metabolites reviewed in Lai and Lan, 2015; Knoot et al., 2018). Finally, biomass derived from cyanobacterial production processes could be used in animal feed supplements or converted into organic fertilizers, especially if cells are engineered for optimal nutritional and nutraceutical content (Singh et al., 2016).

Relative to heterotrophic microbial host species, cyanobacteria possess key limitations that have kept them from being as widely adopted as bioproduction chassis. First, in comparison to many heterotrophic workhorses, such as the bacterium *Escherichia coli* and the yeast *Saccharomyces cerevisiae*, cyanobacteria have relatively slow division rates. In part, this is due to the relative energy density that is contained in solar energy as compared to a rich medium (Zhang, 2011), since the carbohydrates and other organic carbon sources in rich media have high levels of potential energy stored in their bonds (Utschig et al., 2011). The low energy density of sunlight also impacts cyanobacterial autotrophic productivity, and the specific productivity of target metabolites tend to be lower than that of heterotrophic microbes (Nogales et al., 2013). Nevertheless, some species of cyanobacteria have division rates that compete with that of industrial yeasts (Yu et al., 2015; Jaiswal et al., 2018) (see below). Secondly, the number of genetic tools available for cyanobacterial hosts continues to be limited relative to leading heterotrophic model organisms. Additionally, most cyanobacterial species are polyploid (Griese et al., 2011), which can complicate acquisition of fully segregated strains (Kelly et al., 2018), especially in the presence of restriction-modification systems in cyanobacteria that can limit transformation efficiencies (Stucken et al., 2013). Importantly, the technology for growing cyanobacteria at large scales is underdeveloped and low-cost bioreactors or other cultivation platforms systems such as open ponds need to be improved (Knoot et al., 2018). Bioreactors design must contend with the conflicting demands of scaling in two dimensions (to capture sunlight) while minimizing liquid volumes and reactor cost so that operational and capital expenses can be economically viable (Chisti, 2013; Nozzi et al., 2013; Acién et al., 2017). Genetic instability of heterologous pathways can also decrease bioproduction of cyanobacterial strains (Jones, 2014) and is increased by the abundance of repeated DNA motifs that lead to increased homologous recombination in cyanobacteria (Cassier-Chauvat et al., 2016). As outlined below, a number of research efforts have been directed toward overcoming cyanobacterial host limitations in recent years.

### Model Cyanobacterial Strains

While cyanobacteria are an extremely diverse phylum, a relatively small number of cyanobacterial strains have been selected as models, often because these strains have features that mitigate some of the limitations described above (Table 1). Notably, model workhorse cyanobacteria include *Synechococcus elongatus* PCC 7942 (hereafter *S. elongatus* PCC 7942), the first reported strain to be transformed through natural DNA uptake pathways (Shestakov and Khyen, 1970), and *Synechocystis* sp. PCC 6803 (*Synechocystis* PCC 6803), originally isolated in 1968 (Stanier et al., 1971), the first strain to have complex *in silico* models built for genome-scale prediction of metabolism (Fu, 2009). *S. elongatus* has been extensively used as a model of the circadian clock (Ditty et al., 2003), while *Synechocystis* PCC 6803 has served as a useful species for the investigation of core photosynthetic complexes due to its capacity to be grown under photoautotrophic, mixotrophic, or heterotrophic conditions (Vermaas, 1996). *Nostoc* sp. PCC 7120 (*Nostoc* PCC 7120) is a filamentous freshwater cyanobacterial strain which has been used extensively as a model to investigate cellular differentiation (Kumar et al., 2010). *Nostoc* PCC 7120 is capable of fixing nitrogen by forming heterocysts (Cai and Wolk, 1997), which are differentiated cells that efficiently catalyze the reduction of dinitrogen (Herrero et al., 2001), or be exploited for hydrogen production (Tamagnini et al., 2002). These three model species are arguably the best studied strains, but all have relatively modest doubling times: *Nostoc* PCC 7120 (14–15 h) (Callahan and Buikema, 2001), while *S. elongatus* PCC 7942 and *Synechocystis* PCC 6803 have doubling times around 7–12 h (Vermass et al., 1988; Mori et al., 1996). Furthermore, these established models have relatively limited capacity to withstand high light intensities and elevated temperatures that are expected to be encountered in outdoor bioreactors (Yu et al., 2013).

**Table 1:** Common cyanobacterial model organisms.

**Strain**	**Genome size**	**Endogenous plasmids**	**Lifestyle features**	**DNA transfer methods**
*Nostoc* sp. PCC 7120	6.4 Mb	6 plasmids size ranging from 5.6 to 408 kb	Freshwater; filamentous	Conjugation (Wolk et al., 1984), electroporation (Thiel and Poo, 1989)
*Synechococcus elongatus* PCC 7942	2.7 Mb	46 kb plasmid (& 7.6 kb non-essential plasmid)	Freshwater; unicellular	Conjugation (Tsinoremas et al., 1994), natural transformation (Shestakov and Khyen, 1970), electroporation (Marraccini et al., 1993)
*Synechococcus elongatus* UTEX 2973	2.7 Mb	46 kb plasmid	Freshwater; unicellular	Conjugation (Yu et al., 2015)
*Synechococcus* sp. PCC 7002	3.0 Mb	6 plasmids size ranging from 4.8 to 186 kb	Euryhaline; unicellular	Conjugation (Kopka et al., 2017), natural transformation (Stevens and Porter, 1980)
*Synechocystis* sp. PCC 6803	3.6 Mb	7 plasmids size ranging from 2.3 to 120 kb	Freshwater; unicellular	Conjugation (Marraccini et al., 1993), natural transformation (Grigorieva and Shestakov, 1982), ultrasonic transformation (Zang et al., 2007), electroporation (Marraccini et al., 1993)

*Features of the most frequently-used model organisms for studies on cyanobacterial physiology and metabolic engineering. Size of primary genome, endogenous extrachromosomal plasmids, osmotolerance, and routinely utilized genetic transformation methods are reported*.

Driven by the desire for cyanobacterial models that are more amenable for bioindustrial applications, other cyanobacterial strains with much more rapid division times have emerged as important models. Significant efforts have been focused on *Synechococcus* sp. PCC 7002 (*Synechococcus* PCC 7002) in recent years, a unicellular cyanobacterium with a faster doubling time of ~2.6 h (Ludwig and Bryant, 2012). *Synechococcus* PCC 7002 is also capable of growth in a variety of salt, temperature, and light conditions (Sheng et al., 2011; Ruffing et al., 2016), enabling the possibility of utilizing saltwater resources for growth media. More recently, still faster-growing strains have been reported, including *Synechococcus elongatus* UTEX 2973 (*S. elongatus* UTEX 2973) (Yu et al., 2015) and *Synechococcus elongatus* PCC 11801 (*S. elongatus* PCC 11801) (Jaiswal et al., 2018). Interestingly, although the growth rates of these strains are substantially faster (1.5–3 h), genome sequence and proteomic approaches have shown that they are exceptionally closely-related to the much slower-growing model, *S. elongatus* PCC 7942 (Mueller et al., 2017). Indeed, the genome of *S. elongatus* UTEX 2973 is 99.8% identical to *S. elongatus* PCC 7942 and differential regulation of a relatively small subset of common pathways largely accounts for the substantial growth differences (Abernathy et al., 2017; Mueller et al., 2017; Tan et al., 2018). Another related strain, *S. elongatus* PCC 11801 is ~83% identical to *S. elongatus* PCC 7942 and shares some key modifications with that of *S. elongatus* UTEX 2973 that are responsible for its higher growth rates and increased tolerance to certain environmental stresses (Jaiswal et al., 2018). In a recent publication, Ungerer et al. identified five single nucleotide polymorphisms (SNPs) in three genes (*atpA, ppnK*, and *rpaA*) as responsible for rapid growth in *S. elongatus* UTEX 2973 (Ungerer et al., 2018) and these SNPs were also present in *S. elongatus* 11801. The a*tpA* SNP yielded an ATP synthase with higher specific activity, the *ppnK* SNP encoded a NAD^+^ kinase with significantly improved kinetics, and the *rpaA* SNPs caused broad changes in the transcriptional profile. The filamentous cyanobacterium *Leptolyngbya* sp. strain BL0902 (*Leptolyngbya* BL0902) was also recently identified (Taton et al., 2012), can be transformed by conjugation (Taton et al., 2012) and some molecular tools have been characterized (Taton et al., 2014).

Curiously, while *S. elongatus* UTEX 2973 is closely related to *S. elongatus* PCC 7942, it can be only transformed by conjugation (Yu et al., 2015), while *S. elongatus* PCC 11801 is naturally competent for genetic transformation. Apart from *Nostoc* PCC 7120, the other models described above are naturally competent, facilitating the genetic modification of these strains (Porter, 1986; Koksharova and Wolk, 2002). Furthermore, the genomes of the model cyanobacteria described in Table 1 have been sequenced and the information has been organized in the database, CyanoBase (Fujisawa et al., 2017). The access of genome information, together with the construction of metabolic models (see below), makes it possible to both understand the basic metabolism of cyanobacteria and to achieve higher levels of metabolic redirection and control (Lai and Lan, 2015).

## New Tools for Synthetic Biology in Cyanobacteria

In comparison to the genetic toolboxes for work in popular heterotrophic chasses like *E. coli* and *S. cerevisiae*, relatively limited genetic tools have been developed in cyanobacteria (Sun et al., 2018). The design of standardized gene expression parts (e.g., promoters, terminators) that can be modularly recombined with other genetic elements with predictable outputs has catalyzed a revolution in the complexity of heterotrophic genetic circuits (Popp et al., 2017). Unfortunately, many of the synthetic biology tools and modular parts developed for model heterotrophs often do not perform as robustly in the context of cyanobacterial strains (Huang et al., 2010). For example, many characterized *E. coli* promoters do not display similar traits when used in cyanobacteria (Heidorn et al., 2011). These problems may arise in part because of species-dependent distinctions in key features, such as RBS sequences or promoter recognition by endogenous transcription, leading to unpredictability in gene expression (Huang et al., 2010; Wang et al., 2012; Camsund and Lindblad, 2014). In recent years, significant efforts have been made in adapting molecular techniques from other organisms in order to extend cyanobacterial toolboxes and facilitate cellular reprogramming for increased production yields. Here, we outline the latest advances in cyanobacterial synthetic biology tools, including promoters, riboswitches, RBSs, reporters, modular vectors, and markerless selection systems.

### Engineering Promoters to Enhance Protein Expression

Many of the most advanced synthetic biology circuits and pathways today are firmly rooted in an extensive, well-defined library of promoter “parts” that exhibit predictable behaviors when used to drive the expression of a range of target genes (Shetty et al., 2008). By contrast, the number of constitutive and inducible promoters that have been well-characterized in cyanobacteria has been historically small, and considerable variation is often observed in the expression level achieved for distinct heterologous genes. Some limiting factors have compromised the development of cyanobacterial inducible genetic systems, including toxicity of inducers, leaky expression in the absence of inducer, and inducer photolability. Constitutive promoters may also be useful when continuous gene expression is desired, although many endogenous cyanobacterial promoters that are used for this purpose are dynamically regulated by circadian rhythms.

**Figure 1:**
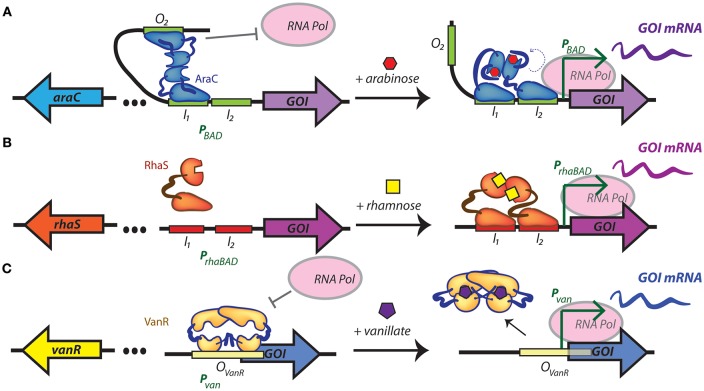
Regulatory mechanisms of inducible promoters recently introduced into cyanobacteria. **(A)** Arabinose-inducible gene expression from P_BAD_, which is both positively and negatively regulated by the transcriptional regulator AraC. In the absence of arabinose, an AraC dimer contacts the O_2_ and I_1_ half sites of the promoter favoring the formation of a DNA loop that is unfavorable for RNA polymerase binding. When arabinose is present, AraC undergoes a structural change that releases the DNA loop, and ideally positions an AraC activation domain that promotes gene transcription. **(B)** Rhamnose-inducible gene expression from P_*rhaBAD*_. In the absence of rhamnose RhaS is unable to dimerize, limiting its capacity to bind to promoter elements. In the presence of rhamnose, a dimer of RhaS binds to the I_1_ and I_2_ repeat half-sites of the *rhaBAD* promoter, recruiting RNA polymerase and activating the transcription of the target gene. **(C)** The vanillate-inducible promoter, P_*van*_, is repressed by a dimer of VanR bound to its operator. When vanillate is present, two molecules bind the VanR dimer, resulting in a conformation change and release of transcriptional repression. I_1_, binding site 1 in P_BAD_ and P_*rhaBAD*_; I_2_, binding site 2 in P_BAD_ and P_*rhaBAD*_; GOI, gene of interest; mRNA, messenger RNA; O_2_, operator of P_BAD_; O_vanR_, operator in P_*van*_; RNA pol, RNA polymerase. In all cases, left and right panel represent uninduced and induced promoters, respectively.

A few recent studies have focused on expanding and characterizing the collection of foreign promoters that can be used to drive gene expression in cyanobacteria. Some inducible promoter elements have been commonly used in selected cyanobacterial models for a number of years, such as the nickel-inducible *nrsB* promoter (Englund et al., 2016; Santos-Merino et al., 2018) or the IPTG-responsive *trc* promoter (P_*trc*_) in *S. elongatus* PCC 7942 or *Synechocystis* PCC 6803 (Geerts et al., 1995; Huang et al., 2010). P_*trc*_ has recently been adapted to other model cyanobacteria, such as *Synechococcus* PCC 7002 (Ruffing, 2014) and *Leptolyngbya* BL0902 (Ma et al., 2014). The L-arabinose-inducible *araBAD* promoter (P_BAD_) that has long been a staple induction system in *E. coli* has recently been introduced in *S. elongatus* PCC 7942 (Cao et al., 2017) and *Synechocystis* PCC 6803 (Immethun et al., 2017). This system is based on the arabinose utilization network, which positively regulates P_BAD_ through the AraC regulator protein (Schleif, 2010) (Figure 1A). Most recently, the use of the rhamnose-inducible *rhaBAD* promoter of *E. coli* has been implemented in the model freshwater cyanobacterium *Synechocystis* PCC 6803 (Kelly et al., 2018) (Figure 1B). Another orthogonal inducible promoter, P_*van*_ from *Corynebacterium glutamicum*, that relies upon vanillate-induced suppression of the repressor VanR has been tested in *S. elongatus* PCC 7942 (Taton et al., 2017) (Figure 1C). Some of these promoters have been translated to faster-growing cyanobacterial strains, such as P_*trc*_ in *Synechococcus* PCC 7002 (Ruffing, 2014) and the IPTG-induced promoter P_*lac*_ in *S. elongatus* UTEX 2973 (Song et al., 2016), although the number of inducible promoters remains relatively limited in the cyanobacterial strains that possess the most promising features for bioproduction.

In some model cyanobacteria, a limited number of constitutive promoters have been characterized relative to one another. For example, P_A2520_ and P_A2579_, two native promoters of *Synechococcus* PCC 7002, have been found to drive strong expression of heterologous genes (Ruffing et al., 2016). Most recently, nine native promoters have been characterized for their expression in *Synechocystis* PCC 6803 to enrich the promoter toolboxes (Liu and Pakrasi, 2018). Other examples of publications that characterize endogenous promoters in cyanobacteria can be found here (Huang et al., 2010; Markley et al., 2015). Yet, in cyanobacteria, many endogenous promoters are strongly influenced by the circadian clock machinery, and therefore they may not be truly constitutive through a 24 h period (Liu et al., 1995; Markson et al., 2013; Camsund and Lindblad, 2014).

Development of synthetic promoters that have been modified for improved expression specifically in cyanobacteria has emerged as another promising strategy. In *S. elongatus* PCC 7942, a tandem promoter composed of a truncated native promoter PR from *rrnA* of *S. elongatus* PCC 7942, and the consensus-σ70 PS promoter from *E. coli* has been designed (Chungjatupornchai and Fa-Aroonsawat, 2014). Zhou et al. reported the development of a constitutive promoter P_*cpc*560_ in *Synechocystis* PCC 6803 that produced a high-level of gene expression (Zhou et al., 2014). It is based on a truncated native promoter (P_*cpcB*_) and its strength resides in the presence of multiple transcription factor binding sites. In another study, P_sca3−2_, a variant of P_*tac*_ of *E. coli* was found to act as constitutive promoter with high levels of expression (Albers et al., 2015). Within the same context of synthetic constitutive promoters, a truncated version of *psbA2* native promoter was developed in *Synechocystis* PCC 6803 (Englund et al., 2016). The expression of this derivate of P_*psbA*2_ increased 4-fold compared to the original. Similarly, inducible promoters can be tuned for better performance in cyanobacteria, such as the anhydrotetracycline-activated variant of P_R40_ from *E. coli*, P_L03_ (Huang and Lindblad, 2013), the IPTG-inducible promoter P_sca6−2_ (Albers et al., 2015), or the T7 RNA Polymerase promoter (Ferreira et al., 2018). Because the promoters described above are modified and/or heterologous, it is possible that they could escape regulatory activities of the circadian clock, yet it is unusual for these promoters to be characterized in more than one circadian period, or at varied light intensities. To solve this problem, some promoters have been characterized for their capacity to drive heterologous gene expression under multiple conditions (e.g., light/dark, aerobic/anaerobic) such as the FNR-activated promoter, P_O2_, described in *Synechocystis* PCC 6803 (Immethun et al., 2016).

### Optimizing Ribosome Binding Sites for Biotechnological Applications

The rate of protein production from a mRNA transcript also depends on the strength of the RBS in recruiting ribosomes for translation. The position and sequence of a given RBS significantly influences translational efficiency. Although “RBS calculators” have long been under development for heterotrophic microbes (Salis, 2011), it is only relatively recently that such efforts extended toward development of RBS libraries for cyanobacteria. RBS sequences from the BioBrick Registry of standard biological parts have been characterized in *Synechocystis* PCC 6803 (Heidorn et al., 2011; Englund et al., 2016). In one recent example, 20 native RBS elements have been characterized in *Synechocystis* PCC 6803 (Liu and Pakrasi, 2018), including two previously described by Englund et al. (2016). These efforts are becoming increasingly coordinated with attempts to develop synthetic RBSs that are based on *in silico* modeling tools for *Synechococcus* PCC 7002 (Markley et al., 2015), *Synechocystis* PCC 6803 (Heidorn et al., 2011; Taton et al., 2014; Xiong et al., 2015; Thiel et al., 2018), or *S. elongatus* PCC 7942 (Taton et al., 2014); (Wang et al., 2018).

Efforts to expand the toolbox of characterized promoter elements are foundational contributions that will enable more sophisticated circuit design in cyanobacteria, yet the capacity to fully predict expression output from a given element remains elusive. Context-specific features of a given expression construct can alter the performance of a given promoter-RBS combination, decreasing the modularity of promoter elements. For example, it is well-established that secondary structure can arise between a specific heterologous gene that can interfere with transcription or translation, leading to variability when expressing different genes from an identical promoter-RBS cassette. Promoter elements that are based on a bicistronic design have been developed for *E. coli* that exhibit much more consistent performance, regardless of the downstream gene sequence that is being expressed (Mutalik et al., 2013). No such system has been described in cyanobacteria and few promoters have been as extensively characterized. Therefore, it often remains difficult to anticipate the likely expression level of a construct during the design phase.

### Riboswitches as Tools for Robust Control of Gene Expression

Riboswitches are versatile tools for genetic engineering and allow control of gene expression by manipulating secondary structure within a mRNA transcript. A riboswitch is composed of an aptamer sequence that imposes a secondary-structural conformation on the mRNA and which influences translational efficiency from the transcript. The aptamer sequence has cis-activating or cis-repressing effects on the mRNA on which it is encoded, while a trans-acting factor can bind and encourage the formation of an alternative secondary-structure conformation (Nudler, 2006). Frequently, the aptamer sequence is designed to form a stem-loop structure that prevents the attachment of ribosomes to a 5′ RBS. A trans-acting factor (which can be a small molecule metabolite or a non-coding regulatory RNA) binds to the aptamer in a manner that stimulates a new conformational state and increases accessibility of the RBS, improving translation of the encoded protein. Riboregulators have features that make them powerful tools for controlling gene expression when used in tandem with transcriptional-based approaches. These include the fact that they typically exhibit a high degree of modularity, can drive the expression of proteins over a large, physiologically-relevant range (thus avoiding issues such as toxicity or inclusion body formation), minimize “leaky” expression (protein production in the absence of inductor), have fast response times (time between ligand binding and protein expression), are tunable (a large dynamic range of protein expression with increasing inducer), and can be used to regulate multiple genes simultaneously (Callura et al., 2010).

Relative to other microbial systems, the use of cyanobacterial riboregulators is just beginning to become more wide-spread and only a few riboswitch designs are in common use for metabolic engineering applications (Connor and Atsumi, 2010). A modified theophylline-dependent synthetic riboswitch was first reported in *S. elongatus* PCC 7942, allowing a strict regulation of protein production in this cyanobacterium (Nakahira et al., 2013). This effective riboswitch has since been implemented in other cyanobacteria such as *Synechocystis* PCC 6803, *Leptolyngbya* BL0902, *Nostoc* PCC 7120, and *Synechocystis* sp. strain WHSyn (*Synechocystis* WHSyn) (Ma et al., 2014; Armshaw et al., 2015; Ohbayashi et al., 2016). Similarly, Taton et al. have also developed NOT gate molecular circuits using transcriptional repressors controlled by theophylline-dependent synthetic riboswitches to downregulate gene expression in five diverse strains of cyanobacteria, including three model organisms, *Nostoc* PCC 7120, *Synechocystis* PCC 6803, and *S. elongatus* PCC 7942, as well as two recent isolates, *Leptolyngbya* BL0902 and *Synechocystis* WHSyn (Taton et al., 2017). A native cobalamin-dependent riboswitch has been reported in *Synechococcus* PCC 7002 (Pérez et al., 2016), although it remains unclear if this genetic tool can be implemented in other cyanobacteria that are not cobalamin auxotrophs. More recently, another native riboswitch, a glutamine aptamer, has been described in *Synechocystis* PCC 6803 (Klähn et al., 2018). However, it exhibited poor ligand affinity compared to the aptamers of other riboswitch classes. These newly-described native regulatory sequences open up the possibility of discovering new cyanobacterial riboswitches still not identified. Future approaches that focus on the combination of *in silico* structural analysis and *in vivo* genetic tuning of riboswitches that can be modulated by a wide variety of ligands hold the promise of contributing significantly to metabolic engineering efforts (Berens and Suess, 2015).

### Reporter Proteins

Reporters allow for easy quantitation of gene expression, visualization of subcellular localization, and interaction of proteins with other cellular components (Berla et al., 2013). Fluorescent reporter proteins do not require additional substrates to be detected *in vivo* and there are an ample range of colors. However, the use of fluorophores can be complicated in cyanobacteria due to the autofluorescence of photosynthetic pigments. The phycobilins and chlorophyll molecules that are major components of the photosynthetic electron transport chain can cause competitive absorbance of excitation light source, re-absorbance of fluorescent or bioluminescent reporter emission, and signal interference (Yokoo et al., 2015; Ruffing et al., 2016). Because of strong chlorophyll autofluorescence, use of red fluorophores is not recommended, but many other reporters including GFPmut3B (a mutant of green fluorescent protein) and EYFP (enhanced yellow fluorescent protein) have been routinely used (Pédelacq et al., 2006; Huang et al., 2010; Yang et al., 2010; Heidorn et al., 2011; Huang and Lindblad, 2013; Landry et al., 2013; Cohen et al., 2014). Despite the frequent use of fluorescent proteins in cyanobacteria and the continual development of improved variants, further improvements to available reporters could greatly increase their range of applications (Rodriguez et al., 2017). Recently developed fluorophores with superior brightness, photostability, and quantum yield have great promise, including mOrange, mTurquiose, mNeonGreen, or Ypet (Chen et al., 2012; Ruffing et al., 2016; Jordan et al., 2017). In addition to fluorescent proteins, luciferase-based bioluminescence assays are routinely used, especially for tracking gene expression patterns throughout the circadian cycle or under different environmental conditions (Fernández-Piñas et al., 2000; Cohen et al., 2015). Luciferase reporters are ideal for monitoring gene expression because of the short half-life of the enzymes, which provides a readout that is close to real-time (Ghim et al., 2010). Alternatively, fluorophores can be modified with protein degradation sequences that greatly reduce their half-life (Wang et al., 2012), increasing their suitability as a transcriptional readout (Noguchi and Golden, 2017).

### Modular Vector Systems for Engineering Cyanobacteria

Vector systems based on the use of standard biological parts (http://parts.igem.org) and assembly schemes (e.g., BioBrick and BglBrick) were originally designed for organisms such as *E. coli, B. subtilis*, or yeast in order to increase the modular assembly of many different parts. By contrast, most standard genetic tools and vectors were developed for a specific cyanobacterial strain and generally, these tools have not been designed to be modular. In recent years, a few vector systems have been designed specifically to work in diverse strains and/or to contain a modular organization, which could facilitate standardization and characterization of component parts.

A notable modular vector system was described in 2014, using a range of plasmids designed to be compatible with a broad host-range (Taton et al., 2014). These plasmids included both autonomously replicating plasmids and suicide plasmids for gene knockout and knockin, and were characterized in diverse cyanobacterial strains to ensure their proper functioning. As a part of this work, the authors also created a web server, CYANO-VECTOR, that can assist in the *in silico* design of plasmids and assembly strategies. Similarly, chromosomal integration vectors carrying standard prefix and suffix sequences suitable for BioBrick-based cloning have been designed for *Synechococcus* PCC 7002 (Vogel et al., 2017) and *S. elongatus* PCC 7942 (Kim et al., 2017). In a recent article, a versatile system called CyanoGate based on the Plant Golden Gate MoClo kit and the MoClo kit for the microalgae *Chlamydomonas reinhardtii* was developed (Vasudevan et al., 2018). Vasudevan et al. demonstrated that the functionality of this system was robust across different two cyanobacterial species, *Synechocystis* PCC 6803 and *S. elongatus* UTEX 2973.

### Markerless Selection as a Tool to Facilitate Cyanobacterial Engineering

In cyanobacteria, as in other bacteria, metabolic engineering involving multiple genetic manipulations requires multiple selective markers. To make deletions, genes are normally replaced by antibiotic resistance markers to evaluate what phenotypic effects take place. However, the generation of a strain with numerous deletions is restricted using this method because the availability of resistance markers is limited. Alternatively, a range of markerless selection strategies has been developed to increase the number of modifications that may be performed to modify genetically these organisms.

The first markerless system described in the cyanobacterium *S. elongatus* PCC 7942 relies on a dominant streptomycin-sensitive *rps12* mutation (Matsuoka et al., 2001). The method is based on a double selection cassette composed of a kanamycin resistance gene and, as an alternative negative selection marker, a *rps12* wild-type copy that confers a dominant streptomycin sensitive phenotype. Streptomycin-resistant, kanamycin-sensitive markerless mutants can be recovered in a second transformation (Takahama et al., 2004). The main drawback of this method is the need to work in a genetic background that contains the appropriate *rps12* mutation. Moreover, this strategy requires two, time-consuming transformation events and cloning of two different suicide vectors. Recently, new time-saving alternatives have been developed for markerless gene replacement in cyanobacteria.

Begemann et al. described a counter-selection method for *Synechococcus* PCC 7002 based on organic acid toxicity (Begemann et al., 2013). The system was based on the use of the product of the *acsA* gene, an acetyl-CoA ligase. The loss of AcsA function was used to develop an acrylate counter-selection method. Another alternative counter-selection method was developed for *Synechocystis* PCC 6803, which involves the use of the endogenous nickel inducible promoter to drive an *E. coli* derived toxin gene known as *mazF* (Cheah et al., 2013). MazF is an endoribonuclease that acts as a global inhibitor for the synthesis of cellular proteins, because it cleaves mRNA at the ACA triplet sequence. A different markerless gene deletion system that only requires a single vector has also been described for *Synechocystis* PCC 6803 and uses a *nptI*-*sacB* double selection cassette (Viola et al., 2014). The *nptI* gene confers resistance to the antibiotic kanamycin, while expression of the *sacB* gene is toxic to bacteria grown on sucrose-containing media. Counter-selection based on *sacB* is not functional in *Synechococcus* PCC 7002 (Zhang and Song, 2018), possibly because *sacB* selection is sensitive to salt (Kunst and Rapoport, 1995) required for growth of this marine cyanobacterium. A few markerless gene deletion systems have been shown to work in multiple cyanobacterial strains, for example Kojima et al. developed an efficient method for generating knockouts in *Synechocystis* PCC 6803 and *Synechococcus* PCC 7002 (Kojima et al., 2016). This system is based on knocking out the *aas* gene, an acyl-acyl carrier protein synthetase, and selecting the mutants by their free fatty acid tolerance.

**Figure 2:**
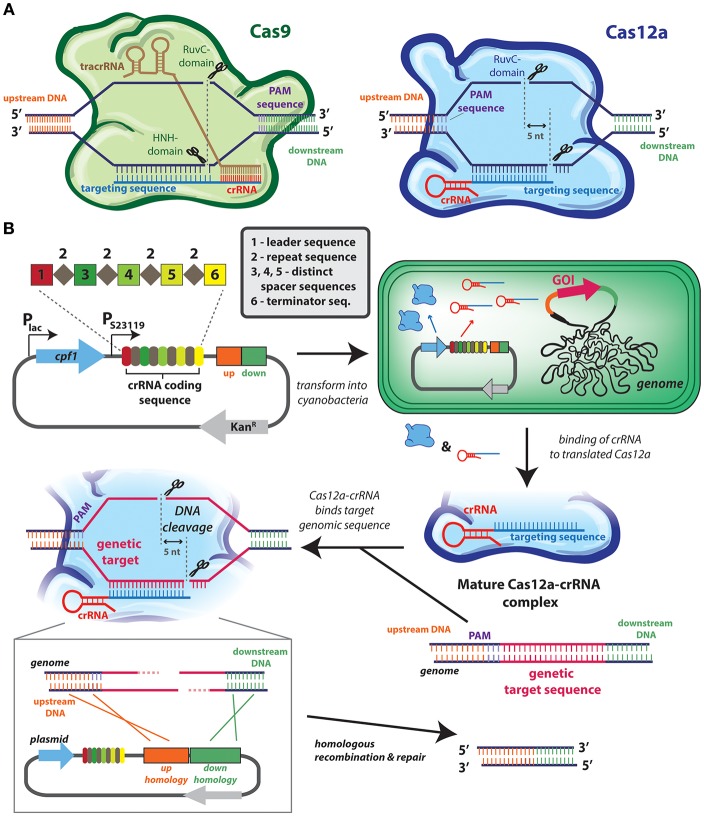
CRISPR-*cas* technologies used for genome editing of cyanobacterial genome. **(A)** CRISPR-*cas* systems used in cyanobacteria: Cas9 (left) and Cas12a (right). In type II systems, Cas9 (green) forms a complex with the crRNA (red) and tracrRNA (brown), whereas in type V systems, Cas12a (blue) forms a complex with its crRNA (red). Both nucleases identify their target sites on the basis of complementarity of the guide sequence (blue) by heteroduplex formation, as well as the presence of a neighboring protospacer-adjacent motif (PAM; purple), although the positioning of the PAM is distinct between systems Cas9 cleaves the complementary DNA strand using its HNH domain, and a second RuvC domain to cleave the non-complementary strand. Cas12a cleaves target DNA using one RuvC endonuclease domain, and a second endonuclease activity is attributed to a novel nuclease domain. **(B)** Example application of CRISPR/Cas12a in *Synechococcus elongatus* PCC 7942. This system is based on the use of a single plasmid that contains: the *cas12a* gene under the *lac* promoter and a crRNA-encoding array (from *Francisella novicida*) under a constitutive (J23119) promoter. This plasmid allows for inducible, transient expression of *cas12a*. This crRNA-encoding array contains a leader sequence (1) followed by direct repeat (2) and spacer (3, 4, 5) sequences, and a terminator (6). In most cyanobacterial CRISPR-Cas reports a single spacer region (e.g., number 3) acts as the genome target spacer, while other spacers are not utilized. Multiple genomic regions can be targeted by encoding other spacers with complementary sequences, or the efficiency of modifying a single genomic loci can be improved by utilizing multiple spacers (Niu et al., 2018). Homology regions designed to promote recombination-mediated repair can also be encoded in the plasmid (represented by orange and green rectangles). Once inside the cell, an active and mature Cas12a-crRNA complex forms and is directed to the target sequence. After the introduction of the 5-nt staggered double-stranded break, the homology regions in the plasmid serve as a repair template to introduce the intended genomic changes. The transconjugants are selected on BG11 agar plates supplemented with kanamycin. The positive colonies are streaked on BG11 agar plates to cure the plasmid and they are assayed to check the loss by the inability to grow on kanamycin-containing media. Depicted system is modeled after the one described by Ungerer and Pakrasi (2016).

The most recent markerless systems are based on CRISPR-based technology (Figure 2) that does not require any counter-selection genes (Behler et al., 2018). In general, all CRISPR-Cas [clustered regularly interspaced short palindromic repeats (CRISPR)/CRISPR-associated protein (Cas) system] technology relies on the capacity to target a protein (typically a nuclease) to a very precise genomic locus. This specificity is conferred by a single-guide RNA (sgRNA) is programmed to be a complement to the target genomic site, and which assembles with Cas9 or Cas12a (formerly known as Cpf1) into an effector complex (Figure 2A). Depending upon the CRISPR system, the effector complex may contain other RNA sequence: the CRISPR-Cas9 system requires two separate RNA strands, the CRISPR RNA (crRNA) that encodes the guide sequence, and trans-activating crRNA (tracrRNA), while CRISPR-Cas12a requires only a single crRNA (Figure 2A). Most cyanobacterial genomes naturally contain CRISPR-Cas repeat sequences (Cai et al., 2013) useful in defending the cell against foreign genetic material as the Cas nuclease can be directed to cleave sequences that are specific to an exogenous source (e.g., viral DNA).

The specificity of Cas9 and Cas12a targeting has been repurposed to design markerless genome editing systems, and other genetic control elements (Li et al., 2016; Ungerer and Pakrasi, 2016; Wendt et al., 2016; Niu et al., 2018; Ungerer et al., 2018; Xiao et al., 2018). When directed to a DNA target within the cyanobacterial genome, the double-stranded DNA breaks induced by the CRISPR effector are lethal unless they can be repaired in a manner that alters the DNA so that it is no longer recognized by the sgRNA (Figure 2A) (Behler et al., 2018). Double-stranded DNA breaks engage cyanobacterial DNA repair machinery, which can resolve the damage by error-prone non-homologous end joining or by homologous recombination if a suitable template is available. To induce an inactivating mutation in a target gene it is often sufficient to express Cas9 or Cas12a along with a gene-specific sgRNA and to rely upon the error in the genetic repair systems to introduce point mutations and frameshifts that will inactivate the gene without the need for a selectable marker. More advanced genome editing (e.g., knockout of specific regions or insertion of new DNA at the target locus) can be accomplished through homologous recombination if a suitable template is also introduced into the cell (see Figure 2B). This markerless methodology has been used to introduce point mutations, knock-out large genomic regions, and “knock-in” genes in a range of cyanobacterial species, including *Synechocystis* PCC 6803 (Xiao et al., 2018), *S. elongatus* UTEX 2973 (Ungerer and Pakrasi, 2016; Wendt et al., 2016), *S. elongatus* 7942 (Li et al., 2016; Ungerer et al., 2018), and *Nostoc* 7120 (Ungerer and Pakrasi, 2016; Niu et al., 2018).

The CRISPR-Cas system is still under active development and some limitations may need to be overcome before it becomes a methodology that completely replaces more traditional cyanobacterial genome engineering techniques. One limitation is that colonies recovered from CRISPR-mediated transformations can have a low penetrance of the desired genomic alteration. For example, early reports of CRISPR editing in cyanobacteria have shown between 20 and 70% of recovered strains are the desired mutant (Li et al., 2016; Ungerer and Pakrasi, 2016; Wendt et al., 2016; Xiao et al., 2018). The low efficiency of some transformations requires screening and validation of a higher number of recovered colonies to obtain the correct strain. It may be possible to overcome this limitation by encoding two spacers that target the genomic region rather than one (see spacer depiction in Figure 2B), as shown in recent report in *Nostoc* PCC 7120 (Niu et al., 2018). The expression of Cas9 also appears to be toxic in a dose-dependent manner in some cyanobacteria, such as *S. elongatus* UTEX 2973 (Wendt et al., 2016), although this toxicity is not apparent in others (Xiao et al., 2018), even in some closely-related species (Li et al., 2016). While the mechanism by which Cas9 causes toxicity remains unclear, it is possible to substitute Cas12a for Cas9 to bypass the issue in many species (Ungerer and Pakrasi, 2016; Swarts and Jinek, 2018). Yet, such uncertainties also contribute to the concern that CRISPR-mediated techniques can lead to alter off-target genomic sites that could lead to misinterpretation of observed phenotypes (Fu et al., 2013). Strategies to minimize off-target CRISPR-Cas activity have been extensively explored in other organisms, but have not been rigorously evaluated in cyanobacteria. Finally, although genomic modifications introduced by CRISPR are themselves markerless, the sgRNA and nuclease themselves are often introduced on plasmids that require selectable markers (Figure 2B), and following genome editing, it is often desirable to cure the plasmid through some form of counter-selection (Xiao et al., 2018). This can make the process of genome editing by CRISPR-Cas considerably longer than traditional selection-based approaches.

CRISPR-based applications reach beyond markerless genome engineering to a wider range of applications. Briefly, nuclease-dead variants of Cas9/Cas12a do not generate DNA breaks, but can still be targeted to specific DNA sequences via sgRNA can be fused to other functional domains (e.g., transcriptional activator or repressor domains). This creates hybrid Cas proteins that bind to endogenous sequences, but which can perform other functions, such as regulating gene expression. Recently, these approaches have been used to create synthetic transcription factors able to modulate the expression of essential genes and key metabolic pathways in cyanobacteria. For a more comprehensive review of CRISPR-based applications in cyanobacteria, see (Behler et al., 2018).

## Improving Carbon Capture and Directing Carbon Flux Through Rational Engineering Approaches

Engineering cyanobacterial strains for bioproduction requires that not only heterologous genes and pathways be expressed in new hosts, but also that carbon that is captured by cyanobacteria be efficiently directed toward the desired metabolic products (Stephanopoulos, 2012; Woo, 2017). Thus, it is a prerequisite for metabolic engineers to optimize pathways to increase the total carbon flux toward target product generation, and/or to enhance the pool size of rate-limiting metabolites (Kanno et al., 2017; Carroll et al., 2018). Although the regulation of carbon partitioning in cyanobacteria is not fully understood, it can be diverted under certain conditions like nutrient deprivation and irradiance stress, and therefore stress conditions are routinely used in studies that aim to increase product yield (Woo, 2017). New predictive tools that can assist in channeling fixed carbon to desired metabolic pathways have been in active development. These tools include models for metabolic analysis and flux balance analysis and will be described in greater detail below. Beyond channeling carbon flux, another strategy is to improve the total pool of available carbon, and therefore multiple efforts to improve carbon fixation rates of cyanobacteria have been pursued.

### Improving Carbon Fixation Rates

Many efforts to engineer cyanobacteria for enhanced productivity have emphasized increasing the total rate of carbon fixation, most frequently by improving the activity or efficiency of RuBisCO. Cyanobacteria have highly efficient carbon concentration mechanisms (CCM) relative to other phototrophs such as algae and plants (Price et al., 2011). The cyanobacterial CCM is efficient in part because it uses bicarbonate transporters to actively transport bicarbonate into the cell, which effectively overcomes the slower (10^4^-fold) diffusion rates of CO_2_ in water compared to air (Price et al., 2011). Ultimately, the accumulated bicarbonate is transported across the carboxysome shell, and converted to CO_2_ in the carboxysome lumen, where RuBisCO is concentrated (Badger et al., 2002; Price et al., 2008). Increasing the efficiency of the cyanobacterial CCM could not only increase the concentration of RuBisCO's substrate, but also reduce the production of energetically-costly photorespiratory byproducts. Recently, extra bicarbonate transporters were expressed in *Synechocystis* PCC 6803, leading to a 2-fold enhancement of the growth rate and a higher amount of biomass accumulation (Kamennaya et al., 2015).

**Figure 3:**
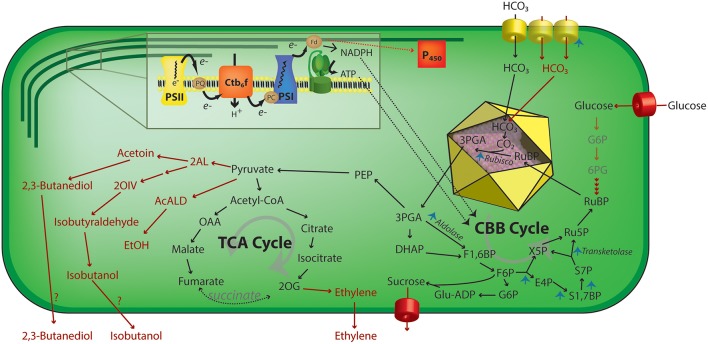
Schematic representation of engineering strategies that increase photosynthetic activity in cyanobacteria. Using ATP and NADPH produced by the light reactions (inset), the CBB acts to assimilate inorganic carbon via a RuBisCO—mediated reaction in the carboxysome (yellow icosahedron) lumen. Upregulation of multiple steps of the carbon concentration mechanism or CBB cycle enzymes has been shown to increase total carbon uptake rates by cyanobacteria (blue arrowheads). Additionally, the regeneration of RuBisCO substrate, ribulose 1,5-bisphosphate, by feeding external carbohydrates has been used to increase CO_2_ assimilation rates. Multiple metabolic pathways (red arrows) that have been heterologously expressed in cyanobacteria have also been demonstrated to increase the effective rate of carbon fixation, perhaps by relieving sink inhibition of photosynthesis. 2AL, 2-acetolactate; 2 OIV, 2-ketoisovalerate; 2OG, 2-oxoglutarate; 3PGA, 3-phosphoglycerate; 6PG, 6-phosphoglycerate; AcALD, acetaldehyde; Ctb_6_f, cytochrome b_6_f; E4P, erythrose 4-phosphate; EtOH, ethanol; F1,6BP, fructose-1,6-bisphosphate; F6P, fructose-6-phosphate; Fd, ferredoxin; G6P, glucose 6-phosphate; Glu-ADP, Glucose-ADP; OAA, oxalacetate; P450, cytochrome P450; PC, plastocyanin; PEP, phosphoenolpyruvate; PQ, plastoquinone pool; PSI, photosystem I; PSII, photosystem II; Ru5P, ribulose-5-phosphate; S1,7BP, sedoheptulose-1,7-bisphosphate; S7P, sedoheptulose-7-phosphate; TCA, tricarboxylic acid; X5P, xylulose-5-phosphate.

Other efforts have focused upon improving carbon fixation rates by balancing the enzymatic activities in the Calvin-Benson-Bassham (CBB) cycle to improve its total metabolic flux. Liang and Lindblad have demonstrated that overexpression of any of four separate enzymes in the CBB cycle (RuBisCO, Sedoheptulose bisphosphatase, fructose bisphosphate aldolase, or transketolase) can improve total carbon fixation rates in *Synechocystis* (Liang and Lindblad, 2016) (Figure 3). Furthermore, modifying the expression of these CBB enzymes could also enhance the rate of heterologous production of ethanol in *Synechocystis* PCC 6803 (Liang et al., 2018). Kanno et al. have recently described a novel strategy to improve carbon fixation rates by focusing upon importing a continuous supply of substrate for RuBisCO. By expressing glucose transporters, they successfully used the oxidative pentose phosphate pathway to convert some imported sugars for the synthesis of D-ribulose 1,5 bisphosphate. Using this approach, they demonstrated that an engineered strain was able to produce a targeted biochemical, 2,3-butanediol, under both light and dark conditions (Kanno et al., 2017).

### Improving Carbon Fixation Through Sink Engineering

While it is frequently assumed that the rate limiting metabolic step of carbon fixation is directly related to the slow catalytic activity of RuBisCO, photosynthetic activity can be limited by other metabolic steps. Utilization of the primary products of the CBB cycle can limit step photosynthesis rather than the fixation of carbon dioxide itself. This is concept was first explored in plant models, where the mechanisms underlying triose phosphate utilization (TPU) limitation are more deeply characterized. In plants, there is a physical separation between where primary products of the CBB are generated (source tissues, e.g., leaves) and where much of these products will ultimately be utilized to support metabolic activity and growth (sink tissues; e.g., roots). Efficient operation of photosynthetic metabolism requires the balance of light energy (“source”) that can be highly dynamic in the environment with an equivalent capacity to utilize/dissipate this energy using anabolic metabolism or quenching mechanisms (“sinks”). Without absorption of adequate photons (i.e., low “source”), a photosynthetic organism will starve; and without sufficient pathways to process or dissipate absorbed energy (low “sink”), end products of photosynthesis can accumulate leading to feedback inhibition and overreduction of the electron transport chain (ETC) (Gifford et al., 1984; Paul and Foyer, 2001). TPU limitation on photosynthesis can occur either because of experimental manipulation (e.g., exogenously supplied carbohydrates, or chemical inhibition of carbohydrate transport/catabolism), or because the sum of metabolic processes downstream the CBB are insufficient to remove triose phosphates at an equivalent rate as they are being generated (Sawada et al., 1986; Sharkey et al., 1986; Krapp et al., 1991; Paul and Foyer, 2001; Adams et al., 2013; Demmig-Adams et al., 2014).

While a number of published reports in plants have sought to enhance biomass accumulation by reducing TPU limitation, relatively few studies exist that suggest that cyanobacterial photosynthesis may also be limited by downstream metabolism. Much of our current knowledge on sink limitation in cyanobacteria is indirect, where researchers have found the activity of a heterologous metabolic pathway expressed in a cyanobacterial model leads to increased photosynthetic activity and/or quantum efficiency (Ducat et al., 2012; Oliver et al., 2013). For example, expression of transporters that allow export of a number of bioproducts in cyanobacteria can lead to increases in photosynthetic activity relative to a parental line that lacks the production pathway. Specifically, some degree of enhanced photosynthetic activity has been in diverse cyanobacterial strains engineered to export sucrose (Ducat et al., 2012), isobuteraldehyde (Li et al., 2014), 2,3-butanediol (Oliver et al., 2013), or ethylene (Ungerer et al., 2012) (Figure 3; red text and arrows).

Our group has recently reported a more comprehensive analysis on the photosynthetic effects that occur following activation of a heterologous carbon sink (i.e., sucrose production and secretion) in *S. elongatus* PCC 7942 (Abramson et al., 2016). In this analysis, we found that photosystem II and photosystem I activities were significantly increased within hours of activating the heterologous sucrose secretion pathway. The quantum efficiency of photosystem II transiently increased following sucrose export and photosystem I activity became less constrained by acceptor-side limitations (i.e., the ability of electron carriers such as ferredoxin to remove the excited electrons generated at the reactive chlorophyll pair of photosystem I; Abramson et al., 2016) and total CO_2_ fixation rates increased (Ducat et al., 2012). Taken together, these results suggest that overall electron flux through the ETC is enhanced following activation of a heterologous export pathway, suggesting that the endogenous metabolism of *S. elongatus* PCC 7942 can be insufficient to completely utilize the products of the light reactions under standard laboratory conditions. Similar studies have shown that heterologous electron sinks also have potential to enhance photosynthesis. When a mammalian cytochrome P450, CYP1A1, was expressed in *Synechococcus* PCC 7002 it was able to utilize reductant from the ETC to catalyze a desired monooxygenation reaction and introduction of this pathway was also associated with an improved photosynthetic efficiency and increased electron flow rate by up to ~30% (Berepiki et al., 2016).

From the above discussion, it appears that at least slower-growing strains of cyanobacteria may be limited by their capacity to utilize products of the CBB cycle. However, it remains unknown if the fastest–growing strains of cyanobacteria (e.g., *S. elongatus* UTEX 2973 and *S. elongatus* PCC 11801) will also exhibit similar increases in photosynthetic flux when engineered to export bioproducts. Current evidence suggests that a greater flux of carbon is allocated away from storage products (e.g., glycogen) and instead invested in cell growth and light-harvesting/carbon-fixation machinery in fast-growing cyanobacteria (Mueller et al., 2017; Zhang et al., 2017; Jaiswal et al., 2018). It is possible that strains with naturally high growth rates will experience less photosynthetic limitations due to over-accumulation of CBB end products, and therefore not exhibit similar enhancements in photosynthesis upon activation of a heterologous metabolic sink.

## Predicting and Engineering Cyanobacterial Metabolism via Genome-Scale Models

Genome-scale models (GSMs) are large-scale stoichiometric models that describe metabolic pathways as stoichiometric coefficients and mass balances of participating metabolites, and are simulated using numerical optimization (Kim et al., 2016). The ultimate goal of these metabolic reconstructions is to give a comprehensive explanation of all biochemical conversions taking place within a living cell or organism, including transport and non-enzymatic reactions (Steuer et al., 2012). Due to ease of implementation and relatively high predictive power, modeling approaches have been used as tools to assist metabolic engineering and production strain development (O'Brien et al., 2015). Computational modeling methods based on the use of GSMs complement experimental research and give a powerful tool to rapidly generate and prioritize testable hypotheses that can be used to guide subsequent experimentation (Dreyfuss et al., 2013). On the other hand, GSMs also provide potential mechanistic explanations for the results obtained in the laboratory (O'Brien et al., 2015).

**Figure 4:**
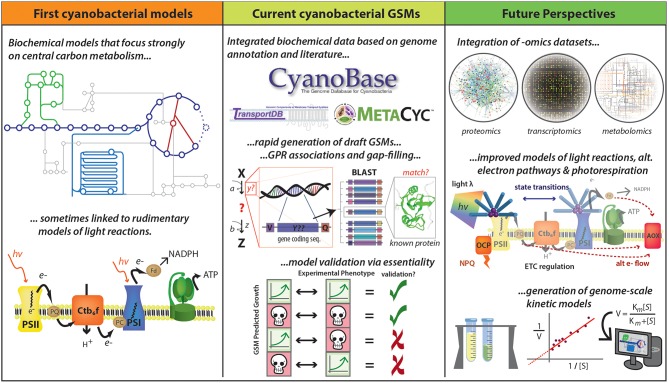
Development of cyanobacterial GSMs. Early cyanobacterial GSMs were developed based on biochemical data and were focused mainly on central carbon metabolism and linked to basic representations of photosynthetic pathways (Cogne et al., 2003; Shastri and Morgan, 2005; Hong and Lee, 2007). Numerous advances have accelerated the reconstruction of GSMs, including improved quantity and quality of data described in public databases (e.g., CyanoBase, TransportDB, KEGG, and MetaCyc) and the development of tools for rapid generation of drafts and for gap-filling. The quality of GSMs has been substantially improved with the inclusion of gene-protein-reaction (GPR) associations, which has been assessed by essential gene prediction based on the availability of experimental data. This methodology allows identify four different kind of predictions: true positives, true negatives, false positives, and false negatives. True positives and true negatives are obtained when both prediction and experimental data indicate a gene is essential and non-essential, respectively (the first and the second case). False positives occur when the model says a gene is essential, but experiments suggest otherwise (the third case). False negatives are generated when a gene is predicted to be non-essential but in reality, it is essential (the fourth case). However, new improvements could further improve GSM predictive ability, including the integration of—omics datasets and the improvement of photosynthesis and photorespiration modeling. A long-term goal would be the development of genome-scale kinetic models, which might be expected to provide more accurate metabolic predictions. Logos for MetaCyc, TransportDB, and CyanoBase are used with permission.

Using the genome sequence of an organism, a draft GSM can be relatively easily compiled (Thiele and Palsson, 2010). Standard procedures have been detailed in the literature, to generate high-quality GSM reconstructions (Feist et al., 2009; Thiele and Palsson, 2010). Moreover, many steps of the reconstruction process have been successfully automated by several software programs (Hamilton and Reed, 2014). This progress has allowed the fast reconstruction of draft GSMs of multiple species (Kim et al., 2016) (Figure 4, Central panel). However, some manual evaluation and curation is required to ensure a high-quality reconstruction (Hamilton and Reed, 2014).

### An Overview of Metabolic Models Developed for Cyanobacteria

A number of GSMs of phototrophic organisms have been published in the last decade, but they are still underrepresented in comparison to heterotrophic microorganisms (Gudmundsson et al., 2017). At the time of this writing, genomes for >270 cyanobacterial species have been sequenced (Fujisawa et al., 2017). However, a limited number of cyanobacterial reconstructions have been made available and preliminary models have only been refined for only a handful of species (Table 2). The earliest metabolic reconstructions were based on biochemical data, focusing mainly on central carbon metabolism and photosynthetic pathways (Figure 4, left panel). However, the recent advances in genome sequencing have allowed the generation of metabolic models at the genome scale. A general naming convention used to describe *in silico* models has been proposed with the form “*i*XXxxx”; where “*i*” refers to an *in silico* model, “XX” are the initials of the person who developed the model and “xxx” the number of genes included in the model (Reed et al., 2003). However, many of the published GSMs do not follow this rule. Moreover, most of them lack the universal metabolite and reaction conventions in the network model. This lack of consistency impedes direct information extraction between different models; maintenance and adherence to a universal standard would greatly improve the updating and curation of GSMs. The inconsistent nomenclature is a key bottleneck in the speed of reconstruction of new high quality GSMs (Kumar et al., 2012).

**Table 2:** GSMs described for cyanobacterial strains.

**Cyanobacteria**	**GSM name**	**Genes**	**Reactions**	**Metabolites**	**% of annotated genes**	**References**
*Arthrospira platensis* PCC 8005	n/a	n/a	121	134	n/a	Cogne et al., 2003
*Arthrospira platensis* C1	*i*AK692	692	875	837	11	Klanchui et al., 2012
*Arthrospira platensis* NIES-39	n/a	620	746	673	24	Yoshikawa et al., 2015
*Synechocystis* sp. PCC 6803	n/a	n/a	70	46	n/a	Shastri and Morgan, 2005
	n/a	86	56	48	2.7	Hong and Lee, 2007
	n/a	633	831	704	20	Fu, 2009
	n/a	337	380	291	11	Knoop et al., 2010
	*i*Syn669	669	882	790	21	Montagud et al., 2010
	*i*Syn811	811	956	911	26	Montagud et al., 2011
	n/a	393	493	465	12	Yoshikawa et al., 2011
	*i*JN678	678	863	795	21	Nogales et al., 2012
	*i*Syn731	731	1,156	996	23	Saha et al., 2012
	*i*HK677	677	759	601	21	Knoop et al., 2013
	*i*SynCJ816	816	1,045	925	25.7	Joshi et al., 2017
	*i*mSyn716	n/a	729	679	n/a	Gopalakrishnan et al., 2018
*Cyanothece* sp. ATCC 51142	*i*Cce806	806	667	587	16	Vu et al., 2012
	*i*Cyt773	773	946	811	15	Saha et al., 2012
*Cyanothece* sp. PCC 7424	*i*Cyc792	792	1.242	1.107	14	Mueller et al., 2013
*Cyanothece* sp. PCC 7425	*i*Cyn731	731	1.306	1.160	14	Mueller et al., 2013
*Cyanothece* sp. PCC 7822	*i*Cyj826	826	1.258	1.110	14	Mueller et al., 2013
*Cyanothece* sp. PCC 8801	*i*Cyp752	752	1.172	994	17	Mueller et al., 2013
*Cyanothece* sp. PCC 8802	*i*Cyh755	755	1.161	973	17	Mueller et al., 2013
*Nostoc* sp. PCC 7120	n/a	n/a	804	777	n/a	Malatinszky et al., 2017
*Synechococcus elongatus* PCC 7942	*i*Syf715	715	851	838	26	Triana et al., 2014
	*i*JB785	785	850	768	29	Broddrick et al., 2016
*Synechococcus elongatus* UTEX 2973	*i*Syu683	687	1,178	1,028	26	Mueller et al., 2017
*Synechococcus* sp. PCC 7002	*i*Syp611	611	552	542	19	Hamilton and Reed, 2012
	n/a	728	742	684	23	Hamilton and Reed, 2012
	*i*Syp708	708	602	581	22.2	Vu et al., 2013
	*i*Syp821	821	744	777	26	Qian et al., 2017

Among cyanobacteria, *Synechocystis* PCC 6803, is the most extensively studied and well-modeled cyanobacterium, with a total of 12 GSMs (Table 2). Network reconstruction is an iterative process and the most robust models are generally created by gradually expanding and updating a prior draft when new data and tools are available (Gudmundsson et al., 2017). This is the case of GSMs: *i*Syn811 (Montagud et al., 2011), *i*Syn731 (Saha et al., 2012), *i*HK677 (Knoop et al., 2013), and *i*mSyn716 (Gopalakrishnan et al., 2018). While distinct cyanobacterial species have unique characteristics, there are common pathways and core carbon metabolic processes that tend to be described in most GSM models. These common pathways can be schematically decomposed as: photosynthesis (to produce ATP and NADPH and fix inorganic carbon), glycolysis (to produce ATP, NADH, and generate precursor metabolites), the citric acid cycle (to produce other precursor metabolites), oxidative phosphorylation (to produce ATP), the pentose phosphate pathway (to produce reducing equivalents and precursor metabolites), carbohydrate synthesis and triacylglycerol synthesis (to build cell walls and store carbon), and inorganic nitrogen assimilation (to produce proteins, DNA, RNA, chlorophyll, and other secondary metabolites using the relevant pertinent precursor molecules) (Baroukh et al., 2015).

Unlike obligate heterotrophic microorganisms, cyanobacteria can utilize light and inorganic carbon, in addition to organic compounds, for the generation of energy and metabolic precursors. The complex mechanisms of light capture makes it difficult to represent it as a simple biochemical reaction in a metabolic networks (Baroukh et al., 2015). Accurate modeling of phototrophic metabolism requires a new level of detail, including modeling the process of light harvesting and electron transport through a variety of possible pathways. Some advances in this area have been achieved in recent years. Nogales et al. proposed a modeling approach for photosynthetic electron flow pathways in detail in *Synechocystis* PCC 6803, including many cyclic electron flow and accessory pathways, enabling the study of photosynthetic processes at the system level (Nogales et al., 2012). In an updated representation of the GSM of *Synechocystis* PCC 6803, the role of photorespiration in cellular growth and the peculiarities of photosynthetic reactions such as light-dependent oxidative stress were integrated (Knoop et al., 2013). Most recently, the development of an approach to incorporate light absorption that factors in the effects of cell shading was achieved in *S. elongatus* PCC 7942 by modeling light as a metabolite (Broddrick et al., 2016). On the other hand, Qian et al. incorporated a light-dependent PSI/PSII electron transport rate algorithm in a GSM of *Synechococcus* PCC 7002, which allowed simulations of photoautotrophic growth at different light intensities (Qian et al., 2017). In a new GSM of *Synechocystis* PCC 6803, an unconstrained photo-respiratory reaction and a mechanism to account for changes in energy absorption from light at different wavelengths have been developed (Joshi et al., 2017). To facilitate a better understanding of respiratory and photosynthetic interactions, these authors included features to model known molecular mechanisms of the photosynthetic network around the thylakoid membrane.

A canonical test useful to benchmark and validate the accuracy of GSMs is to examine their capacity to predict essential genes of the metabolic network (Becker and Palsson, 2008) (Figure 4, central panel).Gene essentiality prediction has been successfully used in several bacteria such as *E. coli* (Suthers et al., 2009; Orth et al., 2011) and *Pseudomonas putida* (Nogales et al., 2008, 2017). The quality of two recent cyanobacterial GSMs has been assessing using gene essentiality datasets: *i*JB785 of *S. elongatus* PCC 7942 (Broddrick et al., 2016) and *i*SynCJ816 of *Synechocystis* PCC 6803 (Joshi et al., 2017). In the first case, 78% of genes were correctly assigned as either essential or non-essential based on data of essentiality *in vivo* obtained from previous dense-transposon mutagenesis experiments (Rubin et al., 2015). In the second case, the new GSM of *Synechocystis* PCC 6803 was able to predict gene deletions with 77% accuracy, based on a qualitative growth comparison of 167 gene-deletion mutants with experimental studies obtained from online databases and a detailed literature search (Joshi et al., 2017).

There are several approaches to improve the accuracy of GSMs. First, the use of data derived from high-throughput growth phenotyping experiments (e.g., knockout mutant strains grown in various media conditions) is very useful for validating and refining metabolic network reconstructions (Gawand et al., 2013). Secondly, integrating of transcriptional regulation has proved to be a vital alternative to build improved models and to investigate the capabilities of reconstructed metabolic networks (Vivek-Ananth and Samal, 2016). Finally, the integration of multi-omics datasets have been used in a number of instances to improve the accuracy of a GSM (Kim and Reed, 2014).

### Tools to Improve GSM Quality and Reconstruction

Manual curation is one the most important steps after the generation of the initial draft of a GSM. The process of manually reconstructing GSMs is complex and requires arduous and time-consuming curation, without which the model quality remains low (Machado et al., 2018). The manual review is required to reflect the true metabolic capabilities of the target organism (Gudmundsson et al., 2017) and relies heavily on experimental, organism-specific information (Thiele and Palsson, 2010). This step involves the examination of the mass and charge balance of individual reactions, gene associations of reactions and reaction directionality (Gudmundsson et al., 2017). This task is typically addressed using the information stored in many public databases such as CyanoBase (Fujisawa et al., 2017), CyanoEXpress (Hernandez-Prieto and Futschik, 2012), KEGG (Kanehisa and Goto, 2000), MetaCyc (Caspi et al., 2006), SEED (Overbeek et al., 2014), BRENDA (Schomburg et al., 2002), and TransportDB (Ren et al., 2004) (Figure 4, central panel). To solve this bottleneck, several tools for rapid automated reconstruction of GSMs are currently available, each offering different degrees of trade-off between automation and human intervention. Some of these tools haven been previously reviewed (Faria et al., 2018; Machado et al., 2018), but only a small number of them have been used in cyanobacteria.

In many cyanobacteria, a high percentage of proteins are annotated as “unknown function” or “hypothetical protein,” e.g., in *Synechocystis* PCC 6803 up to 60% (Lv et al., 2015). Gene-protein-reaction (GPR) relationships define the association between genes, metabolic enzymes, and the biochemical transformations that they perform (Thomas et al., 2014) (Figure 4, central panel). GPR determines the set of metabolic reactions encoded in the genome and provides a mechanistic link between genotype and phenotype (Machado et al., 2016). The inclusion of GPRs within GSMs is essential to improve the quality of GSMs, with the aim of improving their phenotypic predictions (Krishnakumar et al., 2013). New methodologies have been applied to automate the process of adding GPR associations to cyanobacterial GSMs, such as SHARP (Systematic, Homology-based Automated Re-annotation for Prokaryotes). SHARP is a novel PSI-BLAST-based methodology GPR association of the metabolic enzymes involved in prokaryotes, which has been used in cyanobacteria (Krishnakumar et al., 2013). These authors were able to predict 3,781 new GPR associations for the 10 prokaryotes considered, eight of which were cyanobacterial species. These new GPR associations allowed them to annotate gaps in metabolic networks, and to discover several pathways that may be active, thereby providing new directions for metabolic engineering of cyanobacteria.

Another bottleneck during GSM reconstruction is the problem of gap-filling. Metabolic reconstruction via functional genomics (MIRAGE) has been developed specifically to address this problem (Vitkin and Shlomi, 2012), and searches for reactions that are missing from reconstructions purely based on enzyme homology, but where functional genomic data suggests the reactions are present. MIRAGE performance was directly tested by applying it to the reconstruction of a network model for *Synechocystis* PCC 6803, and was then successfully validated against an existing, manually-curated model for this cyanobacterium. Vitkin and Shlomi compared the reconstructed network model for *Synechocystis* PCC 6803 generated by MIRAGE with the manually curated models of Knoop et al. (2010) and *i*Syn811 (Montagud et al., 2011), and found a predictive precision of 70% in the first case and 37.5% for *i*Syn811. MIRAGE was also applied to reconstruct GSMs for 36 sequenced cyanobacteria, including some model cyanobacteria such as *S. elongatus* PCC 7942 and *Synechococcus* PCC 7002 (Vitkin and Shlomi, 2012). However, it has been demonstrated that this methodology was not useful to develop a GSM for the cyanobacterium *Cyanothece* sp. PCC 7424 (Mueller et al., 2013). For example, MIRAGE analysis generated a GSM that contained menaquinone and ubiquinone, compounds shown to not exist within *Cyanothece* PCC 7424 (Collins and Jones, 1981). Moreover, the biomass composition of this GSM did not contain some metabolites that are known to be important components of this species, including lipids, pigments, and cyanophycin. Automated model development tools are useful when there is enough information in the training set of models that they can extract to develop the new one (Mueller et al., 2013). However, some cyanobacterial metabolites are unique, therefore they cannot be detected using this methodology, and require manual annotation.

### Using Flux Balance Analysis for Systems Metabolic Engineering

The mathematical approach most widely used for studying the characteristics and capabilities of large-scale biochemical networks is flux balance analysis (FBA) (Orth et al., 2010). It depends on an assumption of steady-state growth and mass balance (influx equals efflux) (Orth et al., 2010; Qian et al., 2017). FBA calculates the flow of metabolites through a metabolic network, thereby making it possible to predict the growth rate of an organism or the rate of production of a biotechnologically important metabolite (Orth et al., 2010). In FBA simulations, the biomass function is normally used to simulate cellular growth, because it is composed of all necessary compounds needed to create a new cell including DNA, amino acids, lipids, and polysaccharides (O'Brien et al., 2015). However, when FBA is used to simulate the expected rates of production for a metabolite of interest, instead of maximizing the growth rate, it is necessary to maximize the production of this metabolite by optimizing the output flux of the reaction that produces it (Lewis et al., 2012).

FBA and related constraint-based methods can be used to predict the optimal set of gene knockout and overexpression targets to increase the ability of one organism to produce a chemical of interest. With this aim, FBA has been successfully used in GSMs of cyanobacteria to increase the production of several compounds, as summarized in Table 3. This method is a useful alternative when GSMs have little or no kinetic data available for their metabolic enzymes (Orth et al., 2010). Readers may also find more details about FBA results getting in cyanobacteria in this reference (Gudmundsson et al., 2017) and in the references detailed in Table 3.

**Table 3:** Applications of FBA to maximize the production of different compounds in cyanobacteria.

	**Compound**	**Organism**	**Reference**
Alcohols	1-propanol	*Synechocystis* sp. PCC 6803	Yoshikawa et al., 2011
	Butanol	*Synechocystis* sp. PCC 6803	Kämäräinen et al., 2012
	Several alcohols	*Synechococcus* sp. PCC 7002	Vu et al., 2013
	Ethanol, isobutanol, 3-methyl-1-butanol, 2-methyl-1-butanol and propanol	*Synechocystis* sp. PCC 6803	Mohammadi et al., 2016
	Butanol, ethanol	*Synechococcus* sp. PCC 7002	Hendry et al., 2016
	1,3-propanediol, glycerol	*Synechococcus elongatus* PCC 7942	Hirokawa et al., 2017
	Ethanol	*Synechocystis* sp. PCC 6803	Yoshikawa et al., 2017
Bulk chemicals	Isoprene	*Synechocystis* sp. PCC 6803	Saha et al., 2012
	Ethylene	*Synechocystis* sp. PCC 6803	Zavrel et al., 2016
	Succinic acid	*Synechocystis* sp. PCC 6803	Shirai et al., 2016
	Terpenes	*Synechocystis* sp. PCC 6803	Englund et al., 2018
Gases	Hydrogen	*Synechocystis* sp. PCC 6803	Montagud et al., 2010
	Hydrogen	*Cyanothece* sp. ATCC 51142	Saha et al., 2012
	Methane	*Synechococcus* sp. PCC 7002	Comer et al., 2017

### Algorithms Used *in silico* to Improve Cyanobacterial Productivity

Numerous constraint-based methods of GSMs are available to identify the phenotypic properties of an organism and to validate hypothesis-driven engineering of cellular functions toward specific objectives (Kim et al., 2015). In addition to the increasing refinement of higher-quality GSM reconstructions, other computational algorithms have been developed that have expanded the scope, accuracy, and applications for GSMs. Some of these algorithms have been used in cyanobacteria to predict promising gene deletion targets for increased production of target compounds, such as OptGene, minimization of metabolic adjustment (MOMA), OptKnock, and OptORF (Vu et al., 2013; Shabestary and Hudson, 2016). On the other hand, other algorithms have been developed to identify the possible interventions (e.g., up or downregulation of gene expression) that lead to overproduction of a target metabolite (e.g., OptForce) (Shabestary and Hudson, 2016; Lin et al., 2017).

The OptGene algorithm is based on the random implementation of reaction knockouts with the aim to create optimal knockout sets. This algorithm provides the advantage of a high computational speed, enabling solutions to be efficiently reached even for problems of larger size. Additionally, OptGene can optimize for non-linear objective functions, such as the productivity of one specific compound (Patil et al., 2005). This algorithm was recently used to identify knockouts in *Synechocystis* PCC 6803 that improve production of fermentation, fatty-acid, and terpene-derived biofuels (Shabestary and Hudson, 2016). The same authors also used MOMA, an algorithm that predicts the optimal flux distribution of altered metabolism that would require the smallest change from that of wild-type metabolism (Segrè et al., 2002). However, a primary drawback of the MOMA algorithm is that it does not assume optimality of growth or any other metabolic functions (Raman and Chandra, 2009). Despite of these issues, it has been successfully applied in *E. coli* to predict the metabolic phenotype of gene knockouts and to find solutions not detected with FBA (Segrè et al., 2002). Shabestary et al. used MOMA to find knockout strategies that could increase biofuel productivity in *Synechocystis* PCC 6803 (Shabestary and Hudson, 2016). MOMA has been used in *Synechococcus* PCC 7002 to identify knockout mutants to improve chemical production under photoautotrophic and/or dark anoxic conditions (Vu et al., 2013). Shabestary et al. used a third algorithm to find a set of knockouts that led to coupling between biofuel and growth (Shabestary and Hudson, 2016). OptKnock is a powerful algorithm that identifies and subsequently removes metabolic reactions that are capable of coupling cellular growth with chemical production (Burgard et al., 2003). OptORF is an algorithm very similar to OptKnock, but it identifies gene deletions (instead of reaction deletions) and regulatory changes needed to couple growth and chemical production (Kim and Reed, 2010). Nevertheless, the applicability of this algorithm relies heavily on the availability of integrated metabolic and regulatory models, which is not always possible, especially for cyanobacteria (Maia et al., 2016). It was used in *Synechococcus* PCC 7002 to predict metabolic engineering strategies that improve production of both native and non-native chemicals (Vu et al., 2013).

OptForce is an algorithm that identifies all possible engineering interventions by classifying reactions in the metabolic model depending upon whether their flux values must increase, decrease or become equal to zero to meet a pre-specified overproduction target (Ranganathan et al., 2010). This algorithm has been recently extended to include kinetic descriptions for some of the reaction steps (Chowdhury et al., 2014). The new algorithm, k-OptForce, can only be directly applied when kinetic models of cellular pathways are available and unfortunately detailed kinetic models are extremely limited in cyanobacteria (Steuer et al., 2012). OptForce algorithm was applied in *Synechocystis* PCC 6803 to find a set of interventions to couple growth to 1-octanol and limonene production (Shabestary and Hudson, 2016). In the first case, OptForce predicted several reactions in alternative electron flow and required the upregulation of the ferredoxin:NADPH oxidoreductase reaction. In the case of limonene production-growth couple, a significant number of reaction knockouts were required, including some components of the cyclic electron flow and alternative electron flow. In a recent report, Lin et al. used this algorithm in *Synechocystis* PCC 6803 to enhance isoprenoid production (Lin et al., 2017). OptForce predicted several interventions: the up-regulation of two pentose phosphate pathway genes, ribose 5-phosphate isomerase and ribulose 5-phosphate 3-epimerase, and the overexpression of a geranyl diphosphate synthase involved in the limonene biosynthetic pathway. The optimized strain with these modifications demonstrated a 2.3-fold improvement in productivity.

## Conclusions and Perspectives

Cyanobacteria possess desirable characteristics as a chassis for biotechnological production and have demonstrated capacities to produce high-value bioproducts. However, tools from heterotrophs are not always transferrable to these microorganisms, leading to the delay in their advancement as industrial hosts. Accelerating the development of sophisticated genomic tools is likely to greatly assist in making scaled cyanobacterial bioproduction a commercially-viable endeavor. Many recent efforts have been focused on the characterization of new parts and development of libraries and standardized modular parts in cyanobacteria. However, characterization of such parts could likely be significantly accelerated by the development of robust and modular expression libraries, like those that have been established in *E. coli* and yeast. Moreover, the development of well-defined genetic libraries such as genomic, expression, and knockout libraries might facilitate a better understanding of complex phenotypes in cyanobacteria (Ramey et al., 2015). On the other hand, the use of markerless modification systems as well as CRISPR/Cas based technologies could expand and improve the efficiency of genome editing in cyanobacteria. The iterative modification of the genome would allow the assembly of long and complex metabolic pathways, a capacity that is essential for many of the more advanced and ambitious metabolic engineering projects (Ungerer and Pakrasi, 2016; Behler et al., 2018). The development and/or optimization of high-throughput approaches that allow the introduction of several genetic modifications in a single step are needed (Ramey et al., 2015). In *E. coli*, techniques such as Multiplex Automated Genome Engineering (MAGE) (Wang et al., 2009) and trackable multiplex recombineering (Warner et al., 2010) have been successfully used to perform multiple genome engineering modifications in tandem. While some technical challenges would be complicate adoption of such techniques in cyanobacteria, it is notable that MAGE has been successfully applied in combination with CRISPR/Cas technology to engineer polyploid hosts, such as industrial yeast (Lian et al., 2018), and other organisms with more complex genomes, including plants (Sakuma et al., 2014; Hashimoto et al., 2018). The establishment of these approaches in higher organisms, together with the recent development of CRISPR/Cas systems in cyanobacteria, open up the possibility of the development of high-throughput genome engineering of cyanobacterial strains.

The increasing availability of GSMs for different cyanobacterial species have allowed for reiterative design-build-test cycles whereby the predictions from *in silico* models can be validated and improved from experimental outcomes *in vivo*. Recent efforts have focused on the establishment of GPRs to improve the quality of GSMs, and thus, their phenotypic predictions. However, there are still some challenges that need to be addressed to ensure the accuracy of GSM predictions. Improving the scope and accuracy of existing GSMs will likely involve the integration of -omic datasets in to improve *in silico* representation of metabolism and identify additional biological unknowns (Figure 4, right panel). In addition, it is necessary to improve the modeling of photosynthesis, photorespiration, and photodamage (Figure 4, right panel). The correct modeling of photosynthesis and respiratory electron transfer processes might provide some insight into their physiological role in cyanobacteria. Another critical element that is currently absent in GSMs is the incorporation of regulatory pathways that can dynamically alter enzymatic activity and pathway flux (Figure 4, right panel), although in many cases information about regulatory functions is poorly understood in cyanobacteria.

Although existing GSMs have been successfully applied to inform genetic engineering, it is clear that future efforts are being redirected to genome-scale kinetic models, which have still-greater promise. These new models overcome many of GSM shortcomings, such as the lack of representation of metabolite concentrations and enzymatic regulation, which are necessary for a complete physiologically relevant model. Moreover, they enable dynamic analysis of biological systems for enhanced *in silico* hypothesis generation (Srinivasan et al., 2015).

Most efforts in the genetic and metabolic engineering of cyanobacteria for the production of compounds with biotechnological applications have been focused on increasing product yield under laboratory conditions. A limited number of studies has been performed with the aim to optimize cyanobacterial cells to be more compatible with downstream scaled cultivation and processing (Singh et al., 2016; Johnson et al., 2017). The successful utilization of cyanobacterial species for industrial production depends on the development of accurate large-scale cultivation systems. However, most academic researchers lack access to large-scale production systems that are necessary to evaluate the potential of engineered strains under more realistic environmental conditions (Schoepp et al., 2014) and such information is important for the recursive design-build-test process for strain engineering. More communication between academic researchers and industrial partners could assist in the development of accurate equipment to mimic outdoor production conditions at the laboratory scale (as developed in Lucker et al., 2014). In addition to improving product yields, future research should also be addressed to the development of efficient and cost-effective photosynthetic bioreactors (Lau et al., 2015), as well as technologies to harvest the end products (Knoot et al., 2018) to minimize operation costs. Moreover, the high requirements of water and nutrients are other major challenges for economic profitability of large-scale cyanobacterial cultures (Pathak et al., 2018). Further investigation of the capacity of industrial strains of cyanobacteria to grow and remediate wastewater streams might help to alleviate some of these scalability concerns.

## Author Contributions

MS-M, AS, and DD contributed to the writing and editing of the manuscript and generation of figures and tables.

### Conflict of Interest Statement

The authors declare that the research was conducted in the absence of any commercial or financial relationships that could be construed as a potential conflict of interest.
